# The Role of Artificial Intelligence in Diagnostic Radiology

**DOI:** 10.7759/cureus.72173

**Published:** 2024-10-23

**Authors:** Olena Strubchevska, Marko Kozyk, Aleksandra Kozyk, Kateryna Strubchevska

**Affiliations:** 1 Medicine, Jagiellonian University, Krakow, POL; 2 Internal Medicine, Corewell Health William Beaumont University Hospital, Royal Oak, USA; 3 Business, Willamette University, Salem, USA

**Keywords:** artificial intelligence, artificial intelligence in diagnostics, artificial intelligence in radiology, chatgpt, radiology

## Abstract

This article explores the significant impact of artificial intelligence (AI) on radiology through a comprehensive analysis of eight articles published between 2018 and 2024. With the rapid progress of modern science, the diagnostic methods in medicine are subject to change, which creates the need to consider and evaluate new diagnostic techniques such as artificial intelligence. In our study, we will evaluate the diagnostic accuracy of artificial intelligence and radiological image interpretation, as well as the pros and cons of its use and future development prospects in this field. In this article, we also consider the possibility of using GPT-4 for image analysis in radiology. Artificial intelligence is a revolutionary medical tool that can change diagnostic strategies to improve the quality of medical services.

## Introduction

With the increasing global population, which in turn increases the workload of radiologists, the idea of using artificial intelligence to diagnose diseases is becoming increasingly promising. Such a diagnostic strategy could make the work of a radiologist easier and eliminate the possibility of variations in practice since radiologists differ in their ability to recognize and interpret image features. Recent studies show that artificial intelligence can be valuable in radiology as it can assist with image interpretation, critical findings, image quality improvement, prioritization, clinical recommendations, and automated protocol creation [1-5]. In the studies reviewed in this article, artificial intelligence helped diagnose lung diseases, mediastinal diseases, pulmonary embolism, pancreatic cancer [2], etc. The systematic review presented in this article aims to summarize the existing knowledge on using artificial intelligence in radiology based on selected studies.

## Materials and methods

Our primary objective was to determine whether there was statistical significance in the accuracy of this AI model in diagnostics within the field of radiology. Before conducting our study, we reviewed previous works on this or related topics. The results will be summarized in the discussion. To establish our inclusion criteria, we included questions that were only image-based and excluded questions that were text-based. Throughout our study, we analyzed 50 images (n=50), which mainly included X-ray images of the skeletal system and chest with pathologies present. For all images, we used the same question construction model asking to describe the X-ray image and diagnose the disease. It was decided to use a scoring system where correct answers were marked as 1, partially correct -0.5, and incorrect -0. A partially correct diagnosis occurred when the AI identified some aspects of the pathology but missed key details or lacked specificity. This included detecting the general abnormality but not the exact condition or suggesting multiple diagnoses where only one was correct. It reflected useful but incomplete diagnostic information. The results of the analysis were processed and are presented in our results section as a table.

The Preferred Reporting Items for Systematic Reviews and Meta-Analysis (PRISMA) criteria were taken to write this systematic review. The study included articles published between 2019 and 2024 in electronic databases such as PubMed, Google Scholar, Cureus Journal, and the National Library of Medicine. All studies were found by searching for keywords such as "artificial intelligence," "radiological applications," "diseases," and "diagnostics," with the subcategories "radiological image interpretation," "deep learning," and "diagnostic accuracy." An automated keyword-based search strategy was used within the search engines of the databases such as PubMed, Google Scholar, Cureus Journal, and the National Library of Medicine. These built-in search functionalities helped efficiently retrieve relevant studies based on predefined keywords related to artificial intelligence and its applications in diagnostic radiology.

In this research, we utilized a combination of AI-powered diagnostic tools and clinical assessments to analyze patient X-ray images and compare diagnostic accuracy. AI analysis was performed using ChatGPT (GPT-4 model), which interpreted the images and provided preliminary diagnostic feedback based on radiological findings. Also, we would like to mention that all content generated by OpenAI is subject to OpenAI's terms of use and is protected by copyright. According to these terms, we are free to use or share content.

## Results

To conduct this work, we used 50 X-ray images (n=50), which we provided to the ChatGPT for diagnosis. The accuracy of the answer was assessed by comparing the answer provided by the ChatGPT and the description of the X-ray image provided by the specialist. All data remained anonymous.

The responses provided by ChatGPT were analyzed independently by two researchers. All data and analyses from the study were securely recorded in a Microsoft Excel spreadsheet (Microsoft Corporation, Redmond, Washington, USA) and checked for accuracy. We then used automated comparison via Excel to identify any discrepancies between the two records. The researchers then manually checked for any discrepancies. The final results of our researchers’ analysis to determine the accuracy of ChatGPT diagnostics are summarized in Table 1.

**Table 1:** Evaluation of correct answers provided by the ChatGPT

Category	Number of Images (n)	Score 1 (Correct Diagnosis)	Score 0.5 (Partially Correct Diagnosis)	Score 0 (Incorrect Diagnosis)	Average Score	Total Score (out of n)
Skeletal system	25	8	10	7	0.52	13
Chest	25	14	7	4	0.70	17.5
Overall (total)	50	22	17	11	0.61	30.5

Based on the data presented in Table 1, the diagnostic performance of the AI model varies between the two categories of X-rays analyzed: skeletal system and chest X-rays. For the skeletal system, out of 25 images, the AI model correctly diagnosed eight cases, partially diagnosed 10, and made seven incorrect diagnoses. This resulted in an average score of 0.52 and a total score of 13 out of 25.

In contrast, the model performed better with chest X-rays, correctly diagnosing 14 out of 25 images, partially diagnosing seven, and incorrectly diagnosing only four. This yielded a higher average score of 0.70 and a total score of 17.5 out of 25.

When considering the overall performance across the 50 images, the AI model accurately diagnosed 22 cases, provided partial diagnoses for 17, and made 11 incorrect diagnoses. The average score for all images is 0.61, and the total score is 30.5 out of 50. This suggests that while the model shows reasonable accuracy overall, it performs better with chest X-rays compared to skeletal system X-rays.

We can assume that we have obtained such results as a result of the insufficient improvement of artificial intelligence systems and their certain limitations. However, with the rapid pace of their development, these imperfections can soon be eliminated. As you can see, a large percentage were partially correct answers (40% and 28%, respectively). In this case, the ChatGPT usually provided several different answer options, where one turned out to be correct. This confirms that for now, artificial intelligence models require the participation of a person who can confirm the diagnosis. We can also assume that the problems of the ChatGPT with skeletal system images can be explained by the fact that it cannot detect microcracks and minor fractures. In general, the results of this study give hope that in the future, artificial intelligence models, with due improvements, can be used in the field of radiology to facilitate the work of doctors.

Additionally, we provide two examples of diagnostic X-ray images using the ChatGPT below. We have shown the shortcomings of GPT-4 with a simple example demonstrating the need to improve some publicly available artificial intelligence models. Analysis of an X-ray image of a fracture of the fifth metacarpal bone without displacement by GPT-4 revealed that the artificial intelligence could recognize some details of the image but could not make a correct diagnosis. It is worth noting that a radiologist made the diagnosis of a fracture of the fifth metacarpal bone. The image was used in the work with the patient's permission. The patient's data remained anonymous.

According to the ChatGPT, the X-ray image analyzed represented a posteroanterior (PA) view of a left hand. AI stated that all phalanges (finger bones), metacarpals (hand bones), and carpal bones (wrist bones) were visible. It did not find any apparent fractures, dislocations, or bone misalignments.

Possible diagnoses based on the ChatGPT included normal X-ray (AI mentioned that the X-ray might show no pathological findings, especially if taken during routine checks or in cases of mild injury), osteoarthritis, rheumatoid arthritis, and minor fractures. ChatGPT noticed no visible erosions or joint deformities, suggesting early or advanced stages of rheumatoid arthritis. ChatGPT mentioned that in case of recent trauma or minor fractures, especially in the carpal bones or phalanges, the image may require further detailed examination by a radiologist to confirm micro-fractures.

**Figure 1:**
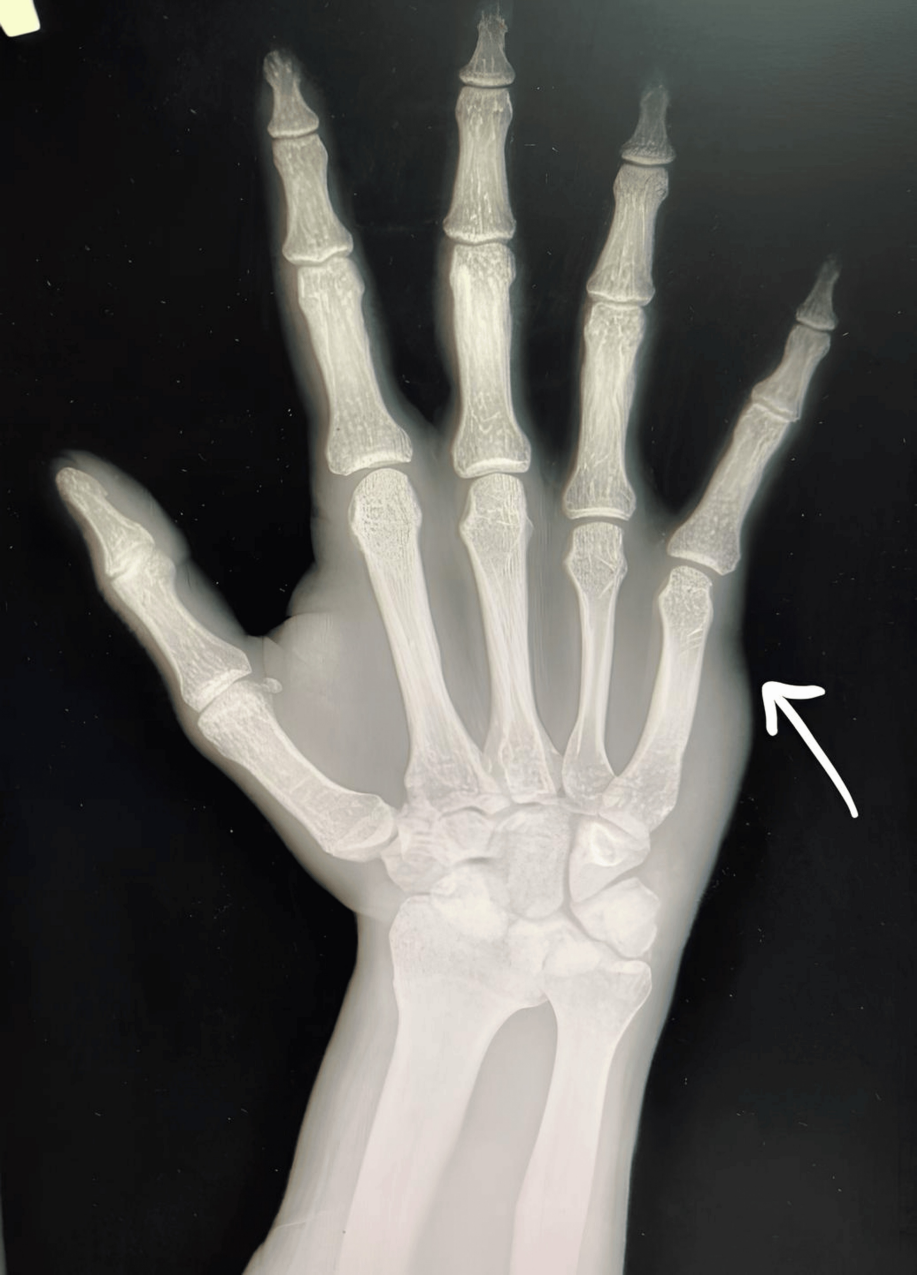
Fracture of the fifth metacarpal bone (arrow)

As we can see, several options were suggested by the artificial intelligence during the diagnostics. The last option was correct, but here we can see that the diagnostics of microtraumas require the intervention of a radiologist since artificial intelligence has difficulties in diagnosing injuries of this type. A more detailed x-ray image is shown in Figure 1.

Several factors come into play when evaluating the pros and cons of ChatGPT's ability to provide medical diagnostics, such as interpreting X-rays or suggesting potential diagnoses. While AI can assist in many aspects of medical care, there are essential limitations and strengths to consider. As we know, ChatGPT can process and provide preliminary diagnostic suggestions in a matter of seconds, which is valuable for professionals needing quick guidance or a second opinion. Also, it has access to vast amounts of medical knowledge and can offer a wide range of information on various conditions. However, ChatGPT is not a medical professional and cannot replace the expertise of trained clinicians. While it can provide general information, it lacks the depth and nuance that comes with years of medical training, patient interaction, and clinical decision-making.

**Figure 2:**
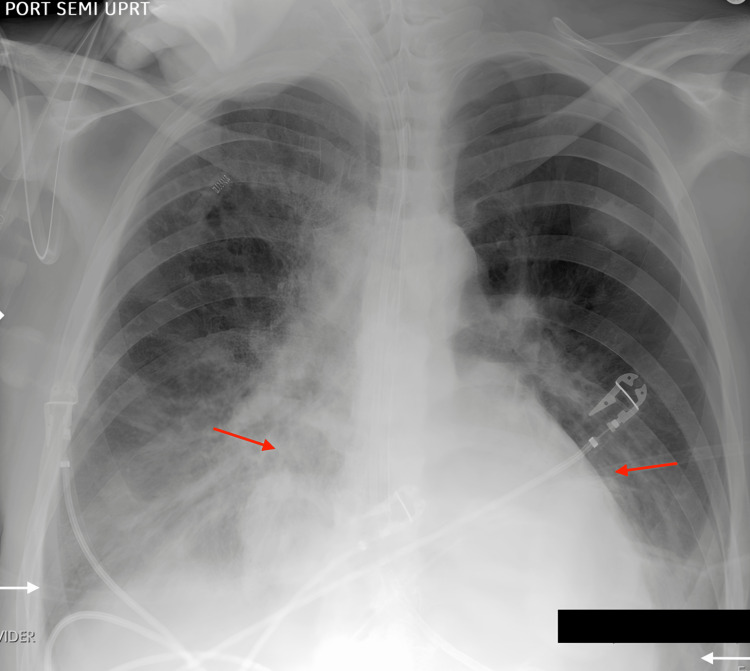
Bibasilar effusions with associated atelectasis or airspace disease Red arrows indicate the borders of the heart, and white arrows indicate costophrenic angles.

Also, we would like to show you another example of an attempt to diagnose an X-ray by ChatGPT. Here, we used an X-ray of a patient with bibasilar effusions with associated atelectasis or airspace disease and enlarged cardiomediastinal silhouette (Figure 2). The patient's data remained anonymous, and the X-ray image was used with the patient's permission.

ChatGPT stated that the X-ray showed potential pulmonary congestion or pneumonia, with diffuse opacification primarily in the right lung. According to it, the heart appeared enlarged, suggesting cardiomegaly, which could indicate congestive heart failure (CHF).

Upon comparing the initial diagnosis provided by ChatGPT with the correct diagnosis of bibasilar effusions with associated atelectasis or airspace disease and an enlarged but stable cardiomediastinal silhouette, we could say that ChatGPT mentioned pulmonary congestion, which indicates fluid buildup in the lungs. This somewhat overlaps with the idea of effusions, although effusions specifically refer to fluid in the pleural space rather than within the lung tissue itself. Also, it suggested the possibility of pneumonia based on the appearance of infiltrates. However, this was inaccurate, as bibasilar effusions and atelectasis were the leading causes of the abnormalities. AI correctly identified the enlarged cardiomediastinal silhouette which aligned with our diagnosis.

While GPT-4 identified key issues like cardiomediastinal enlargement and lung abnormalities, the specific nature of the lung issues (effusion vs. congestion, atelectasis vs. pneumonia) was incorrectly interpreted. This highlights the complexity of X-ray interpretation and the need for precision in recognizing subtle differences between conditions like effusion, atelectasis, and pneumonia.

When discussing potential variations across different AI models, it is important to acknowledge that the diagnostic accuracy and performance of ChatGPT could vary depending on the version or model used. For instance, ChatGPT Plus, which operates on GPT-4, offers improved reasoning abilities and contextual understanding compared to the basic version of GPT-3.5. This could potentially lead to more accurate diagnoses in radiology, as the more advanced model may better interpret subtle details in X-rays and medical imagery.

Moreover, exploring other GPT models available on the OpenAI platform, such as GPT-4-turbo, may reveal differences in processing speed, accuracy, or the ability to handle more complex medical cases. Each model's architecture and optimization can impact how well it performs in specific tasks, particularly when it comes to interpreting medical images where precision is crucial. In addition, comparing alternative AI engines, such as Perplexity AI, could also offer valuable insights. Perplexity, for example, is designed to excel in information retrieval and generating concise, factual responses. By acknowledging these possibilities, the study gains a broader perspective on AI's capabilities in radiology, suggesting that future research could explore how different models perform in comparison to ChatGPT, offering a more comprehensive understanding of AI-driven diagnostics.

## Discussion

A number of studies were conducted from 2018 to 2024 on the use of artificial intelligence in radiology [1-13]. Aggarwal et al. aimed to check deep learning efficiency in identifying pathologies in medical imaging [1]. To accomplish this task, several studies were analyzed (82 studies in ophthalmology, 82 in breast disease, and 115 in respiratory disease were included for meta-analysis). The primary outcome measures included diagnostic accuracy, study design, and reporting standards in the literature. Random-effects meta-analysis showed high area under the curve (AUC) in a variety of domains: in ophthalmology (0.933 to 1) for diagnosing diabetic retinopathy, age-related macular degeneration, and glaucoma; in respiratory imaging (0.864 to 0.937) for diagnosing pulmonary nodules and lung cancer; and in breast imaging (0.868 to 0.909) for diagnosing cancer using different modalities. High heterogeneity across studies and significant differences in methods may overestimate the accuracy of deep learning algorithms in medical imaging. This study states a need to develop specific Enhancing the Quality and Transparency of Health Research (EQUATOR) guidelines for artificial intelligence, especially Standards for Reporting Diagnostic Accuracy (STARD), to address critical questions in this domain.

The study by Higaki et al. [4], published in the Japanese Journal of Radiology, explores the application of deep learning methods to enhance CT and MR image quality. The results showed that deep learning algorithms can significantly reduce noise and improve image clarity without compromising diagnostic accuracy. This improvement is critical to increasing the reliability of radiological assessments and potentially reducing the need for repeat scans. The study highlights the potential and promise of AI-based methods to optimize image quality, thereby contributing to better clinical outcomes.

AI methods for image enhancement have already reached the market or are about to be released. These methods effectively enhance the quality of medical images, allowing radiologists to improve their interpretation accuracy. By reducing noise and enhancing contrast, AI-assisted image enhancement makes it easier to detect subtle abnormalities that might have otherwise been missed in conventional imaging techniques. AI technologies for disease detection are progressing rapidly. They show considerable potential for improving early detection of various diseases, including cancers [2] and cardiovascular conditions, by analyzing imaging data more quickly and accurately than traditional methods.

Overall, the results demonstrate the growing influence of AI in medical imaging, with the potential to transform various aspects of radiology practice. While AI has made notable strides in image enhancement and disease detection, other areas, such as lesion segmentation, diagnosis, treatment selection [6], response assessment, and clinical prediction, remain under development. These advancements suggest that AI has the potential to significantly improve diagnostic accuracy, optimize treatment decisions, and enhance patient care. However, more research and clinical validation are required to integrate these technologies into routine practice fully.

Another study by Jyotsna et al. [7] describes the growing importance of artificial intelligence in radiology, especially regarding image analysis using ChatGPT. AI is promising in improving diagnostic accuracy and efficiency and reducing human error, especially in image analysis and reporting tasks. However, limitations include the need for significant data to train AI systems, the potential for bias, and the importance of human supervision to ensure accuracy. The balance between the potential of AI and the needs of radiologists emphasizes the complementary role of technology rather than its complete replacement of human professionals.

Although still in development, AI tools will likely complement radiologists by providing an additional layer of precision in identifying abnormal tissue or lesions, reducing false positives and negatives, and ensuring more reliable diagnostics. AI is also making strides in diagnostic applications, where AI models are trained to identify and classify diseases based on imaging data. These tools can assist radiologists by providing second opinions or as a rapid triage system [8], ensuring that suspicious cases are flagged for further analysis [9]. Although these technologies are not yet fully integrated into clinical practice, their development is progressing rapidly, and their diagnostic accuracy is continually improving as AI models are trained on more extensive and more diverse datasets.

The study by Lin et al. [10], published in Frontiers in Neuroscience, explores the use of convolutional neural networks (CNNs) to predict Alzheimer’s disease from MRI images in patients with mild cognitive impairment (MCI). The study demonstrates that the CNN model can effectively differentiate between MCI patients whose Alzheimer’s disease progresses and those who do not. This early prediction capability is critical for timely intervention and treatment and can significantly facilitate radiologists' work. The study highlights the promise of using AI to improve early diagnosis and treatment outcomes for Alzheimer’s disease through advanced MRI image analysis.

The study by Rajpurkar et al. published in PLoS Medicine examined the performance of the deep learning algorithm CheXNeXt [11]. They compared the algorithm’s performance in diagnosing diseases from chest radiographs to that of practicing radiologists. The study has important implications for assessing how AI can match or potentially outperform human experts in the accuracy of diagnosing common chest diseases. The study found that CheXNeXt performed on par with radiologists in most conditions, demonstrating a powerful ability to detect pneumonia, pleural effusion, and atelectasis. This suggests that AI could make radiologists’ jobs easier, especially when diagnosing certain diseases. One of the critical contributions of this study is its rigorous comparison, which shows that AI, particularly deep learning, can serve as a reliable tool in diagnostic radiology, potentially improving the efficiency and accuracy of clinical diagnoses. The study also discusses the implications of such technology, suggesting that AI can be an auxiliary tool in clinical settings, helping reduce diagnostic errors and manage large volumes of radiographs. However, the authors also note limitations, such as the study's retrospective nature and the need for further validation in different clinical settings.

The study by Rubin, which was published in the National Library of Medicine (2019) [12], is an in-depth study of the potential of artificial intelligence (AI) in the field of radiology, particularly as it relates to diagnostic imaging. This study highlights the transformative potential of AI technologies and discusses their integration into clinical practice. The main objective of this article is to outline specific ways of developing the use of artificial intelligence in the field of radiology. The emphasis is on ensuring that the benefits of using this technology outweigh the potential risks. It identifies several key areas where AI significantly contributes, such as image acquisition, image analysis, and workflow optimization. The study highlights the ability of AI algorithms, especially deep learning models, to identify patterns and abnormalities in medical images, which is essential for identifying conditions such as cancer, fractures, and neurological disorders. The study also describes the potential of AI in diagnostic optimization, as it can assist radiologists by prioritizing cases that require immediate attention, thereby reducing diagnostic time and potentially improving patient outcomes. Also, this study presents the primary areas of clinical application for artificial intelligence (AI) methods, indicating their development status and market availability. For instance, image enhancement is either currently available or will soon be on the market, reflecting its active implementation in clinical settings.

In contrast, disease detection, lesion segmentation, and diagnosis are still under development, showcasing advancements in AI's capacity to analyze medical images. Additionally, treatment selection, response assessment, and clinical prediction are also in development, highlighting the potential for AI to aid in treatment decisions and predictive analytics in healthcare. Additionally, AI tools can assist radiologists in interpreting complex cases, resulting in more consistent and accurate diagnoses. However, the study does not ignore some of the challenges that must be addressed to successfully implement AI in clinical settings. These challenges include issues related to the quality and quantity of data, the need for reliable validation of AI algorithms, and issues of interpretability and transparency of AI decisions. AI still makes diagnostic errors, so it is not a trustworthy tool. In addition, the study highlights the importance of integrating AI with existing workflows to enhance the role of radiologists rather than replacing them. The study raises issues of maintaining patient privacy, data security, and potential bias in AI algorithms. This paper also calls for developing clear regulatory frameworks to ensure the safe and effective use of AI technologies in clinical practice.

The following study by Tang [13], examines how AI revolutionizes medical imaging by improving image analysis, increasing diagnostic accuracy, and streamlining clinical workflows. Published in Insights into Imaging, the study highlights the capabilities and potential of AI, particularly machine learning and deep learning, in detecting complex patterns in medical images that can aid in the early diagnosis of diseases such as cancer. AI is also known for its promise to automate routine tasks that it could efficiently perform, thereby reducing the workload of radiologists and increasing the efficiency of the overall workflow. However, the study also addresses some challenges associated with using AI, including the need for large, high-quality datasets and concerns about algorithmic bias and data privacy. The study concludes that ongoing research and clear guidelines are essential to fully realizing AI's potential in medical imaging.

Despite the promising advantages of artificial intelligence (AI) in radiology, several limitations hinder its full integration into clinical practice. One significant concern is the risk of algorithmic bias, where AI systems may perpetuate existing disparities in healthcare due to training on non-representative datasets​. Additionally, there are concerns regarding the lack of transparency in AI decision-making processes, making it challenging for radiologists to understand the rationale behind certain outputs.

In conclusion, many studies have been conducted on the use of artificial intelligence methods in radiology [1-13]. These approaches are extremely promising since they can facilitate radiologists' work and improve diagnostic accuracy and image quality [14]. Using these findings, we created the tables presented below (Tables 2, 3).

**Table 2:** Summary of key studies on AI in diagnostic radiology AI: artificial intelligence, CNN: convolutional neural network, EQUATOR: Enhancing the Quality and Transparency of Health Research, STARD: Standards for Reporting Diagnostic Accuracy.

Study	Key Focus	Key Findings
Rajpurkar et al.(2018) [11]	AI in chest radiograph diagnosis	The CheXNeXt deep learning algorithm performed on par with radiologists in diagnosing chest conditions, showing potential to reduce diagnostic errors and handle large volumes.
Nishio et al. (2018) [14]	AI in lung nodule classification	Deep learning successfully classified lung nodules into benign, primary lung cancer, and metastatic cancer, demonstrating AI's potential to enhance lung cancer diagnostics.
Higaki et al. (2019) [4]	AI in image quality enhancement	Deep learning significantly improved CT and MRI image quality by reducing noise, enhancing clarity, and preserving diagnostic accuracy.
Lin et al. (2018) [10]	AI in Alzheimer’s disease prediction	CNNs predicted Alzheimer's progression from mild cognitive impairment, showing promise in early diagnosis and intervention for Alzheimer’s through advanced MRI analysis.
Jyotsna et al. (2023) [7]	GPT-4 Vision for radiological image analysis	GPT-4 Vision can potentially improve diagnostic accuracy and efficiency, particularly in image analysis, though data bias and training limitations need to be addressed.
Tang (2020) [13]	The role of artificial intelligence in medical imaging research	The study underscores the capabilities and potential of AI, especially machine learning and deep learning, in identifying intricate patterns in medical images, which can facilitate the early diagnosis of diseases like cancer.
Rubin (2019) [12]	Artificial intelligence in imaging: the radiologist’s role	The study emphasizes the significance of incorporating AI into current workflows to strengthen the role of radiologists instead of substituting them. It also addresses concerns regarding patient privacy, data security, and the potential biases present in AI algorithms.
Aggarwal et al. (2021) [1]	Diagnostic accuracy of deep learning in medical imaging: a systematic review and meta-analysis	The study indicates the necessity of creating tailored EQUATOR guidelines for artificial intelligence, particularly STARD, to address critical issues in this field.

**Table 3:** Advantages and challenges of AI in diagnostic radiology AI: artificial intelligence.

AI Application	Advantages	Challenges
Chest radiograph diagnosis (CheXNeXt)	Improved diagnostic accuracy, reduced radiologist workload	Needs further validation, potential for data limitations
Lung nodule classification	Accurate classification of lung nodules into different categories	Requires extensive training data, validation in clinical settings
Image quality enhancement	Significant reduction of noise, improved image clarity	Integration with existing radiology workflows
Alzheimer’s disease prediction	Early diagnosis through advanced MRI image analysis	Need for large datasets, concerns over generalizability
GPT-4 Vision for radiology	Enhanced image analysis, potential for automating routine tasks	Data bias, algorithmic transparency

## Conclusions

The study provides critical insights into the use of artificial intelligence in radiology, specifically focusing on its application in image enhancement, disease detection, lesion segmentation, diagnosis, treatment selection, and clinical prediction. Convolutional neural networks (CNNs) are designed to automatically detect patterns within images by learning features such as edges, textures, and shapes, which make them highly effective for tasks like disease detection, image classification, and segmentation. Through multiple layers, CNNs can learn to recognize complex patterns, such as irregularities in tissues or specific disease markers (e.g., tumors or fractures), enhancing diagnostic accuracy. The findings suggest that AI has significantly improved in several areas, such as image enhancement and disease detection. This indicates that radiologists are beginning to integrate AI technologies into clinical practice, potentially improving diagnostic accuracy and efficiency. However, there are still challenges in adopting and applying AI for more complex tasks, such as accurate diagnosis, treatment selection, and clinical prediction. This highlights the need for continued research and innovation to refine AI algorithms and ensure clinical reliability.
